# Stereotactic Body Radiation Therapy for Locally Progressive and Recurrent Pancreatic Cancer after Prior Radiation

**DOI:** 10.3389/fonc.2018.00052

**Published:** 2018-03-07

**Authors:** Philip Sutera, Mark E. Bernard, Hong Wang, Nathan Bahary, Steven Burton, Herbert Zeh, Dwight E. Heron

**Affiliations:** ^1^UPMC Hillman Cancer Center, University of Pittsburgh School of Medicine, Pittsburgh, PA, United States; ^2^Department of Radiation Medicine, University of Kentucky, Lexington, KY, United States; ^3^Department of Biostatics, University of Pittsburgh, Pittsburgh, PA, United States; ^4^Department of Medical Oncology, University of Pittsburgh Cancer Institute, Pittsburgh, PA, United States; ^5^Department of Surgical Oncology, University of Pittsburgh, Pittsburgh, PA, United States

**Keywords:** pancreatic cancer, stereotactic body radiation therapy, locally progressive, reirradiation, overall survival

## Abstract

**Introduction:**

Pancreatic adenocarcinoma is an aggressive malignancy that has consistently demonstrated poor outcomes despite aggressive treatments. Despite multimodal treatment, local disease progression and local recurrence are common. Management of recurrent or progressive pancreatic carcinomas proves a further challenge. In patients previously treated with radiation therapy, stereotactic body radiation therapy (SBRT) is a promising modality capable of delivering high dose to the tumor while limiting dose to critical structures. We aimed to determine the feasibility and tolerability of SBRT for recurrent or local pancreatic cancer in patients previously treated with external beam radiation therapy (EBRT).

**Materials and methods:**

Patients treated with EBRT who developed recurrent or local pancreatic ductal adenocarcinoma treated with SBRT reirradiation at our institution, from 2004 to 2014 were reviewed. Our primary endpoints included overall survival (OS), local control, regional control, and late grade 3+ radiation toxicity. Endpoints were analyzed with the Kaplan–Meier method. The association of these survival endpoints with risk factors was studied with univariate Cox proportional hazards models.

**Results:**

We identified 38 patients with recurrent/progressive pancreatic cancer treated with SBRT following prior radiation therapy. Prior radiation was delivered to a median dose of 50.4 Gy in 28 fractions. SBRT was delivered to a median dose of 24.5 Gy in 1–3 fractions. Surgical resection was performed on 55.3% of all patients. Within a median follow-up of 24.4 months (inter-quartile range, 14.9–32.7 months), the median OS from diagnosis for the entire cohort was 26.6 months (95% CI: 20.3–29.8) with 2-year OS of 53.0%. Median survival from SBRT was 9.7 months (95% CI, 5.5–13.8). The 2-year freedom from local progression and regional progression was 58 and 82%, respectively. For the entire cohort, 18.4 and 10.5% experienced late grade 2+ and grade 3+ toxicity, respectively.

**Conclusion:**

This single institution retrospective review identified SBRT reirradiation to be a feasible and tolerable treatment strategy for patients with previous locally progressive or recurrent pancreatic adenocarcinoma.

## Introduction

Pancreatic adenocarcinoma is an aggressive malignancy that has consistently demonstrated poor outcomes despite advances in surgical techniques, systemic therapy, and radiation techniques. Best outcomes are observed in those who receive surgical resection as it is the only potential for cure. Unfortunately, <20% of patients are deemed resectable at time of diagnosis. Additionally, systemic chemotherapy and radiation play an important role as adjuvant treatment, or definitive treatment in unresectable patients (1). Despite aggressive multimodal treatment, local disease progression and recurrence are common (2–4). Management of locally recurrent or progressive pancreatic cancer has proven to be further challenge as conventional radiation techniques are unable to give further curative doses, surgery is often not an option, and systemic therapy may have been exhausted.

Following recurrence or disease progression, treatment options for local disease control remain limited. Reresection has been demonstrated to be safe and feasible, however, only a small proportion of patients are deemed to be candidates (3, 5). Additional radiation therapy is also limited due to the presence of critical normal structures. These include small bowel, kidneys, and spinal cord and further radiation would cause unacceptable toxicity.

Recently, there has been interest in stereotactic body radiation therapy (SBRT). SBRT is a highly precise modality that is capable of delivering a high biological effective dose while minimizing dose to surrounding tissue (6). The precision afforded by SBRT thereby minimizing dose to critical structures is promising for reirradiation. Prior retrospective reports have identified reirradiation as a potential treatment option (7, 8). Herein, we report the use of SBRT for recurrent and locally progressive pancreatic adenocarcinoma treated in patients with prior radiation therapy.

## Materials and Methods

### Patient Population

Following approval of our institution review board protocol, patients with histologically proven pancreatic adenocarcinoma between 2004 and 2014, who were treated with external beam radiation followed by SBRT, were reviewed. Patients with resectable, borderline resectable, unresectable, medically inoperable, and recurrent tumors were included in this study. Patients were excluded if they had distant metastasis. SBRT was performed on either a CyberKnife Robotic Radiosurgery (Accuray Inc., Sunnyvale, CA, USA) or other linear accelerator-based platforms (Trilogy, TrueBeam) (Varian Medical Systems, Palo Alto, CA, USA). Patient variables including age, race, gender, SMAD4 mutation, surgical status, chemotherapy treatment, prior external beam radiation therapy (EBRT) and SBRT dose, dosimetry, and late toxicity were collected.

### Definition of Parameters

Local, regional, and distant progressions were determined based on radiographic findings on follow-up and/or confirmatory biopsy if done. Local progression was identified as progressive disease (PD) using RECIST 1.1 criteria which is characterized by at least a 20% increase in the sum of diameters of the tumor and a minimum of a 5 mm increase (9). Regional failure was defined as disease progression to the regional nodes defined as n1, n2, or n3 by the JPS classification (10, 11) (or new tumor growth within the pancreas outside of the radiation field). Late toxicity (>3 months post-SBRT) was retrospectively graded with the Common Terminology Criteria for Adverse Events Version 4.0. Patients included in the study had fiducials placed before CT-simulation to assist with target delineation during treatment. Matching was done to both fiducials and soft tissue anatomy depending on the extent of fiducial migration, if any occurred. Patients were simulated in the supine position using four-dimensional CT-scan with IV contrast in a vacuum lock bag and wingboard. The 4D-CT scan was obtained utilizing 1.25 mm slices simulated in a vacuum lock bag. During the time of simulation, a motion study was performed during which we obtained multiple images during the respiratory cycle using the abdominal marker as a surrogate for the respiratory cycle. The signal detected from the abdominal surrogate was used to bin the CT images, creating a series of separate CT scans for each phase in the breathing cycle. We then contoured the gross tumor volume (GTV) to see if any motion was detected during the breathing cycles. If the motion was found to be more than 5 mm, respiratory gating was used. In this technique, we determined which phases of the breathing cycle limit the tumor motion to 5 mm and treat during those specific phases (12). During the patient’s treatment, an equivalent abdominal surrogate signal is used to control the beam on time of the linear accelerator. The GTV was determined based on the simulation CT scan and diagnostic CT scan. For patients treated on a linac, the planning target volume (PTV) margin was added to be approximately 3 mm from GTV with editing off of the bowel. No PTV margin was used for patients treated on CyberKnife. The max dose to the small bowel was limited to 25 Gy for SBRT. The max dose for the kidneys, liver, and cord were limited to 15, 50, and 15, respectively. Multi-fraction SBRT was delivered every other day.

### Endpoints

Our primary endpoints included overall survival (OS), time to local failure, time to regional failure, and late grade 3 or greater radiation toxicity.

### Statistical Analysis

Continuous variables were summarized with median and inter-quartile range (IQR). Categorical variables were summarized with frequency and percentage. The survival endpoints (OS, time to local progression, time to regional progression, and time to distant progression) were analyzed with the Kaplan–Meier method. The association of these survival endpoints with risk factors was studied with univariate Cox proportional hazards models. To build multivariable Cox models for the survival endpoints, the stepwise variable selection was performed. All the variables from univariate models that had a *p*-value of <0.1 were included as potential predictors. Variables were removed from the multivariable model if the *p*-value >0.05. All *p*-values reported are two-sided. The effect of factors on grade 2+ and grand 3+ toxicities was analyzed with logistic regression models.

## Results

### Patient Characteristics

**Figure 1:**
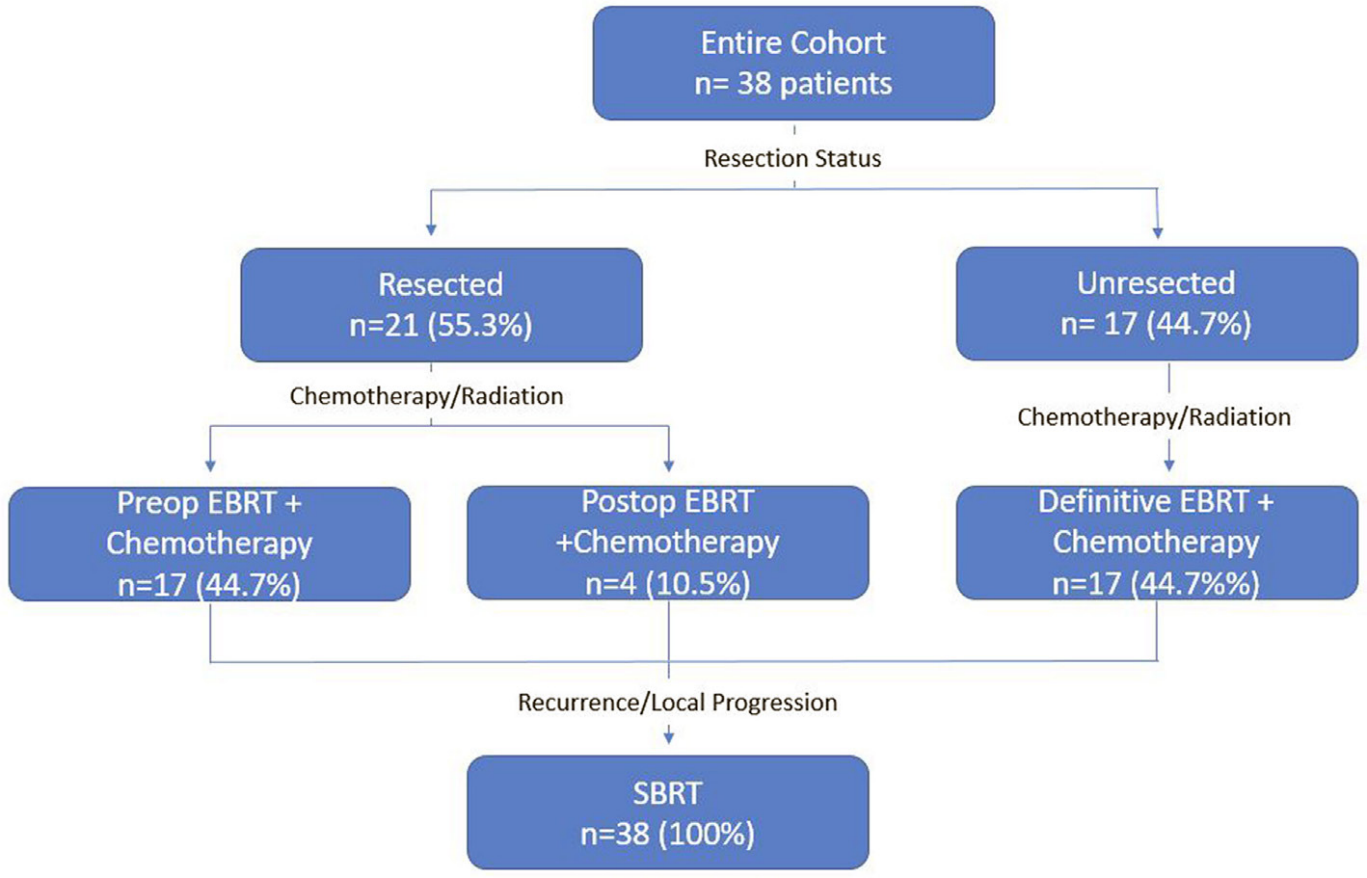
Treatment scheme.

A detailed list of patient characteristics can be found in Table 1. We identified 38 patients with pancreatic adenocarcinoma with prior radiation therapy treated with SBRT for recurrent (42.1%) or locally PD (57.9%). Median age at diagnosis was 69.0 (range 42.7–84.8) with 50% female and 50% male. Location of the tumor within the pancreas included the head (53%), body (11%), uncinate process (11%), neck (8%), and tail (5%). Multifocal disease within the pancreas was seen in 13%. All patients received EBRT with a median dose of 50.4 Gy (IQR, 30–50.4). Surgical status at diagnosis included resectable (55.3%), borderline resectable (7.9%), and unresectable (36.8%). Surgical resection was performed on 52.6% of patients prior to SBRT and 2.6% of patients following SBRT. All patients who received resection underwent a Whipple procedure. Chemotherapy regimens included gemcitabine alone (51%), gemcitabine + capecitabine (14%), gemcitabine + other additional chemotherapy (17%), and 5Fu-based chemotherapy regimens (14%). A treatment scheme can be seen in Figure 1.

**Table 1:** Patient characteristics.

Characteristics	Value (*n* = 38 lesions)
Age (years, range)	42.7–84.8
**Gender**	
Female	19 (50%)
Male	19 (50%)
**CA19-9 value [median value, inter-quartile range (IQR)]**	
At diagnosis	141.4 (90.3, 365.2)
Pre-stereotactic body radiation therapy (SBRT)	85.9 (20.7, 366.9)
Post-SBRT	89.3 (14.7, 458.6)
Change in CA19-9	2.2 (−15.0, 120.2)
**Location**	
Body	4 (11%)
Heal	20 (53%)
Tail	2 (5%)
Uncinate	4 (11%)
Neck	3 (8%)
Genu	0 (0%)
Multiple	5 (13%)
**Resectable status**	
Resectable	21 (55.3%)
Borderline resectable	3 (7.9%)
Unresectable	14 (36.8)
**Surgery**	
Yes	21 (55.3%)
No	17 (44.7%)
Previous external beam radiation therapy dose (median, range)	50.4 (14, 55.8)
Prior fx (median, range)	28 (8, 30)
Prior dose/fx (median, range)	1.8 (1.8, 3)
**Treatment platform**	
Trilogy	15 (39%)
CyberKnife	16 (42%)
Truebeam	7 (18%)
**Chemotherapy**	
Gemcitibine	18 (51%)
Gemcitibine + capcitabine	5 (14%)
Gemcitibine + other	6 (17%)
FU based	5 (14%)
Other	1 (3%)
Gross tumor volume (cc) (median, IQR)	13.7 (8.8, 19)
Planning target volume (cc) (median, IQR)	14 (9.4, 19)
**Fractionation**	
Single	18 (47%)
Multi-fraction	20 (53%)
Dose (median, IQR)	24.5 (24, 30)

### SBRT Treatment Characteristics

In a median of 5.8 months (IQR, 4.1–12.4) after EBRT, SBRT was delivered by Trilogy (39%, *n* = 15), Truebeam (18%, *n* = 7), or CyberKnife (42%, *n* = 16) in either 1 fraction (47%, *n* = 18) or multiple fractions (53%, *n* = 20). Patients received either 22 or 24 Gy in 1 fraction or 36, 30, or 27 Gy in 3 fractions. One patient received 20 Gy in 2 fractions while all others received 3 fractions if treated in multiple fraction regimen. For the entire cohort median, GTV was 13.7 cm^3^ (IQR 8.8–19) and PTV was 14 cm^3^ (IQR 9.4–19).

### Overall Survival

**Figure 2:**
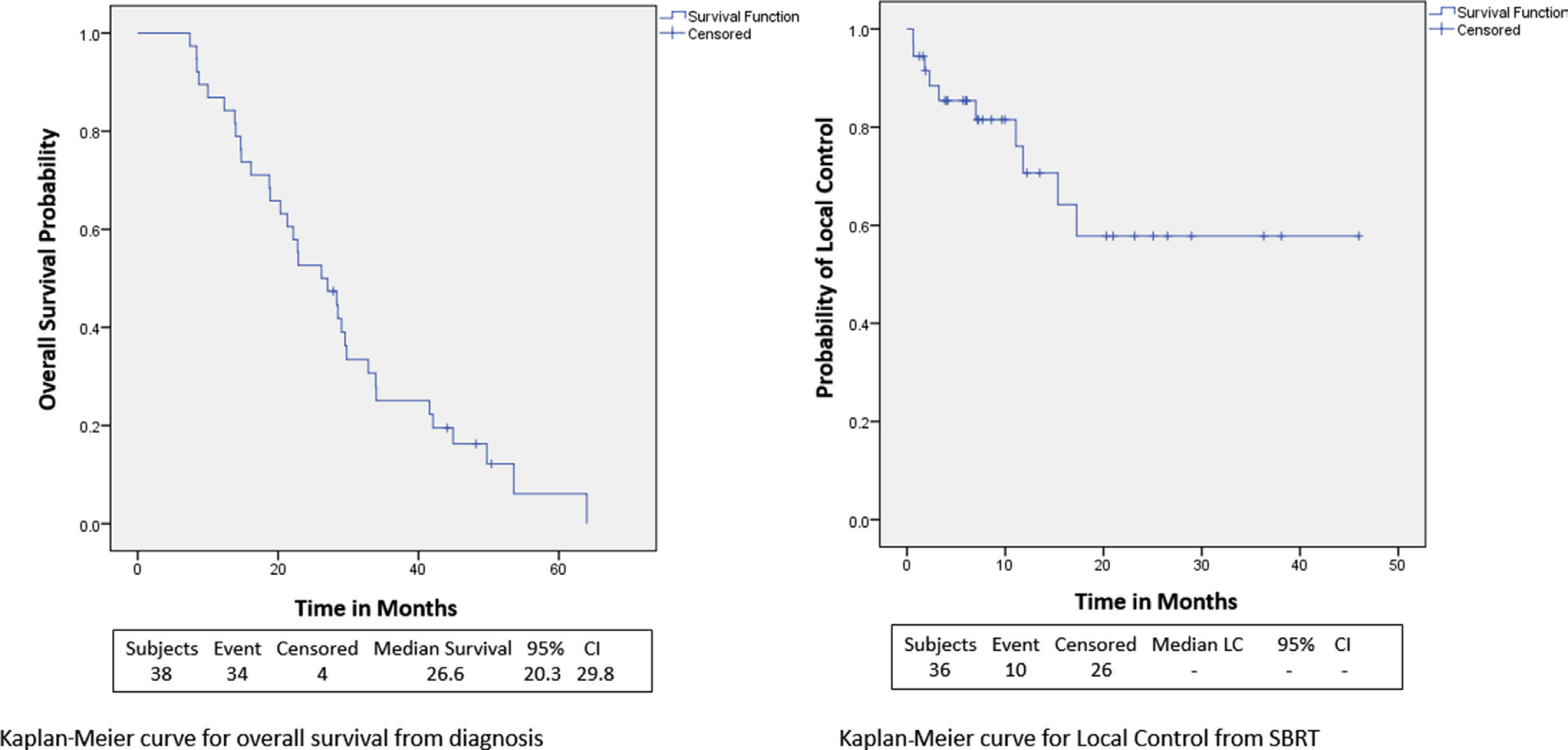
Kaplan–Meier curves for overall survival and LC.

Within a median follow-up of 24.4 months (IQR, 14.9–32.7 months), the median OS from diagnosis for the entire cohort was 26.6 months (95% CI: 20.3–29.8) with 1- and 2-year OS of 87 and 53.0%, respectively (Table 2; Figure 2). Univariate and multivariate analysis demonstrated superior OS significantly associated with recurrent lesions [*p* = 0.0140, HR 0.00007 (95% CI, 0–0.14)], those who received surgery [*p* = 0.0053, HR 0.00007 (95% CI, 0–0.58)], and GTV volume [*p* = 0.0288, HR 0.87 (95% CI, 0.77–0.99)] and inferior OS associated with pre-SBRT CA19-9 [*p* = 0.0102, HR 1.01 (95% CI, 1.002–1.018)] (Table 3). Median follow-up from SBRT was 7.4 months (IQR, 3.4–18.4) with a median survival from SBRT of 9.7 months (95% CI, 5.5–13.8).

**Table 2:** Kaplan–Meier estimates.

Kaplan–Meier estimates	All cohort
**Median follow-up**	
From diagnosis—months (inter-quartile range)	24.4 (14.9–32.7)

**Median survival from diagnosis**	
Median survival—months (95% CI)	26.6 (20.3–29.8)
12-months	87%
24-months	53%

**Median survival from stereotactic body radiation therapy (SBRT)**	
Median survival—months (95% CI)	9.7 (5.5–13.8)
12-months	44%
24-months	20%

**Local control from SBRT**	
Median time to LF (95% CI)	Median not reached (15.4-infinity)
12-months	71%
24-months	58%

**Regional control from SBRT**	
Median time to RF (95% CI)	Median not reached (N/A)
12-months (95% CI)	82%
24-months (95% CI)	82%

**Distant metastases from SBRT**	
Median time to DM (95% CI)	20.2 (9.7-infinity)
12-months (95% CI)	67%
24-months (95% CI)	49%

**Table 3:** Univariate and multivariate analysis of overall survival from diagnosis.

Factor	Hazard ratio (95% confidence interval)	*p*-Value
**Univariate analysis**		

Age	0.97 (0.94, 1.00)	0.0639
CA19-9 at diagnosis	0.9999 (0.9995, 1.0003)	0.5764
Pre-stereotactic body radiation therapy (SBRT) CA19-9	1.001 (1.000, 1.002)	0.0499
Post-SBRT CA19-9	1.0002 (0.9998, 1.0006)	0.3775
Change in CA19-9	1.00074 (0.99994, 1.00154)	0.0712
SMAD4 mutated vs not	1.51 (0.81, 2.82)	0.1944
Location: heal vs body	1.25 (0.36, 4.37)	0.7313
Location: tail vs body	1.12 (0.11, 11.37)	0.9207
Location: uncinate vs body	1.27 (0.27, 5.96)	0.7583
Location: neck vs BODY	2.44 (0.46, 12.96)	0.2956
Location: multiple vs body	1.68 (0.39, 7.29)	0.4880
Location: genu vs body	No data	–
Prior external beam radiation therapy dose	0.99 (0.95, 1.02)	0.4843
Modality: CyberKnife vs triology	1.00 (0.47, 2.13)	0.9151
Modality: truebeam vs triology	0.95 (0.34, 2.67)	0.9152
Recurrent lesion being treated	0.38 (0.18, 0.80)	0.0108
Gross tumor volume (GTV)	1.04 (1.01, 1.06)	0.0026
Planning target volume	1.02 (0.99, 1.04)	0.2131
Multiple fractions	1.12 (0.56, 2.26)	0.7430
Chemo: gemcitibine + capcitabine vs gemcitibine	1.02 (0.34, 3.05)	0.9773
Chemo: FU based vs gemcitibine	0.93 (0.30, 2.92)	0.9001
Surgery	0.27 (0.13, 0.57)	0.0005
Dose	0.96 (0.90, 1.03)	0.2462

**Multivariate analysis**		

Recurrent lesion being treated	0.00007 (0, 0.14)	0.0140
GTV	0.87 (0.77, 0.99)	0.0288
Surgery	0.00007 (0, 0.058)	0.0053
Pre-SBRT CA19-9	1.010 (1.002, 1.018)	0.0102

### Local Control, Regional Control, and Distant Metastases

1- and 2-year freedom from local progression is 71 and 58%, respectively for the entire cohort. Univariate analysis demonstrated inferior 2-year local control significantly associated with post-SBRT CA19-9 (*p* = 0.0243) and recurrent lesions (*p* < 0.0367) (Table 4). No multivariable model was found for local control with the stepwise variable selection method. 1- and 2-year freedom from regional control rates were both 82%. None of the variables analyzed were found to be significantly associated with inferior regional control on univariate or multivariate analysis (Table S1 in Supplementary Material). At 1 and 2 years, the Kaplan–Meier estimated rate of freedom from distant metastasis was 67 and 49%, respectively. Univariate analysis identified pre-SBRT CA19-9 (*p* = 0.0219) and GTV volume (*p* = 0.0137) associated with increased distant metastases and prior EBRT dose (*p* = 0.0314) associated with decreased distant metastases (Table S2 in Supplementary Material). No multivariable model was found for distant metastasis with the stepwise variable selection method.

**Table 4:** Univariate and multivariate analysis of local control.

Factor	Hazard ratio (95% confidence interval)	*p*-Value
Univariate analysis		
Age	0.98 (0.92, 1.04)	0.4550
CA19-9 at diagnosis	0.99996 (0.99946, 1.00046)	0.8816
Pre-stereotactic body radiation therapy (SBRT) CA19-9	1.00191 (0.99991, 1.00392)	0.0616
Post-SBRT CA19-9	1.0015 (1.0002, 1.0028)	0.0243
Change in CA19-9	1.004 (0.998, 1.010)	0.1798
SMAD4 mutated vs not	2.16 (0.44, 10.65)	0.3443
Location: heal vs body	0.36 (0.07, 1.92)	0.2335
Location: tail vs body	0.43 (0.04, 4.83)	0.4920
Location: uncinate vs body	0.21 (0.02, 2.40)	0.2117
Location: neck vs body	0.62 (0.05, 7.10)	0.6979
Location: multiple vs body	0.00 (0.00, infinity)^a^	0.9939
Location: genu vs body	No data	–
Prior external beam radiation therapy dose	0.95 (0.89, 1.01)	0.1297
Modality: CyberKnife vs triology	0.82 (0.18, 3.69)	0.8004
Modality: Truebeam vs triology	2.19 (0.47, 10.15)	0.3156
Recurrent lesion being treated	4.31 (1.09, 16.94)	0.0367
Gross tumor volume	1.03 (0.99, 1.07)	0.1405
Planning target volume	0.97 (0.88, 1.07)	0.5741
Multiple fractions	1.64 (0.45, 5.94)	0.4522
Chemo: gemcitibine + capcitabine vs gemcitibine	2.86 (0.48, 17.06)	0.2490
Chemo: FU based vs gemcitibine	4.02 (0.67, 24.23)	0.1289
surgery	0.97 (0.23, 4.06)	0.9648
Dose	1.00 (0.89, 1.13)	0.9684

*^a^The hazard ratio is 0 because the group defined by value 1 of the variable did not have a local progression event*.

### Late Radiation Toxicity

For the entire cohort, 18.4 and 10.5% experienced late grade 2+ and grade 3+ toxicity, respectively. None of the variables analyzed were found to be associated with grade 2+ or grade 3+ toxicity on multivariate or univariate analysis (Table S3 in Supplementary Material). One patient experienced grade 4 toxicity which was duodenal stenosis requiring urgent operative intervention. Three patients experienced grade 3 toxicity which included nausea (*n* = 2) and enteritis (*n* = 1).

## Discussion

This retrospective review aimed to determine the role of SBRT for recurrent or locally progressive pancreatic adenocarcinoma in patients with prior radiation therapy. As previously noted, despite multi-modality treatment, local control remains poor (13–16). Following recurrence or local progression, management of pancreatic adenocarcinoma becomes considerably more challenging. Following initial resection (if the tumor is resectable) and chemoradiation, very few options remain to provide further local control after disease progression. Systemic chemotherapy remains critically important, as distant metastasis is a major cause of mortality. However, local control is necessary to minimize risk for distant failure (17–20). Local progression can have a significant effect on quality of life causing significant pain and obstruction. Additionally, up to 30% of patients with pancreatic cancer die with locally destructive pancreatic cancer (21). We now show that reirradiation with SBRT in 1–3 fractions with 24–36 Gy is a feasible treatment strategy with acceptable rates of toxicity for recurrent/progressive pancreatic cancer.

As expected, our results identified surgery associated with improved OS on multivariate analysis. As demonstrated in numerous other reports, surgery continues to be essential for prolonged survival (11, 22, 23). Strangely, we also identified recurrent lesions to be significantly associated with improved OS. This, however, is likely a result of patients who develop recurrent disease previously had responsive or stable disease for an extended period prior to recurrence suggesting a favorable histology. Patients considered to have locally PD, progressed soon after initial treatment indicating a more aggressive tumor biology. As our analysis for OS was from diagnosis, the extended survival seen in patients with recurrence was likely a result of their prolonged time from initial treatment to SBRT. We also show pre-SBRT CA19-9 associated with inferior survival. These results contrast previously reported results. Sutera et al. and Herman et al. previously identified post-SBRT but not pre-SBRT CA19-9 associated with worse survival (12, 24). Notably, however, neither of these studies analyzed SBRT for recurrent/locally PD with prior radiation. It is possible CA19-9 values after initial treatment are most prognostic as an indicator of tumor response. In our results, CA19-9 values after initial treatment were pre-SBRT. In the other studies, post-SBRT CA19-9 represented CA19-9 values following initial treatment. Notably, our results did not identify improved outcomes with multi-fractionation as has been previously identified (12, 25). This was likely due to our smaller sample size unable to detect this difference.

Koong et al. previously reported on 23 patients (52.2% received resection) with locally recurrent pancreatic adenocarcinoma after prior EBRT treated with SBRT in 1 or 5 fractions. Median OS from diagnosis was 27.5 and 8.5 months from SBRT. Cumulative local, regional, and distant failure at last follow-up was 19, 14, and 64%, respectively. Following SBRT, 26.1% of patients developed grade 2+ and 8.7% developed grade 3+ toxicity (7). Dagoglu et al. reported on 30 patients (50% received resection) with prior EBRT or SBRT treated with reirradiation SBRT in a median of 25 Gy in 5 fractions for recurrent pancreatic cancer. Median OS from SBRT was 14 months. The 1- and 2-year local control were both 78%. Late grade 3+ toxicity was observed in 7% of patients (8). Finally, Lominska et al. reported on 28 patients (29% received resection) with unresectable locally recurrent or progressive pancreatic cancer with prior EBRT treated with SBRT reirradiation. Patients received a median of 22.5 Gy in 3 fractions as either boost (36%) or salvage (61%) treatment. Median OS from SBRT was 5.9 months. 1-year survival from SBRT and freedom from local progression was 18 and 70%, respectively (26). Our data demonstrate similar results with a median OS from diagnosis and SBRT of 26.6 and 9.7 months, respectively. Additionally, we have shown similar rates of local control with 1-year local progression-free survival of 71%. Finally, like the above studies, we have acceptable or similar rates of late grade 3+ toxicity of 10.5%.

As noted, surgical reresection remains the other primary method of local control following disease recurrence/progression. This, however, has limited utility as very few patients are candidates for this treatment. Miyazaki et al. retrospectively reviewed 170 patients with recurrent pancreatic cancer who received prior resection. Among this cohort, only 11 patients (6.5%) received a second resection. Patients who received a second resection were found to have a significantly greater median OS from diagnosis (78.2 vs 20.3 months, *p* < 0.001). Median survival from reresection/diagnosis of recurrence was also significantly greater in patients receiving a second resection (25.0 vs 9.3 months, *p* < 0.01) (3). This review did not assess rates of surgical complications. Although these are quite impressive results, it is evident that surgical reresection is an excellent option for recurrent pancreatic cancer but only in a select few patients. Considering <20% of patients can undergo an initial resection and only 6.5% of recurrent patients who previously received resection received a second resection, this is not a treatment option for most patients. SBRT appears to be a significantly less selective treatment option able to provide local disease control to a greater proportion of patients. Notably, one patient in our cohort went on to receive surgical resection following SBRT.

The present study adds to the currently literature of management of recurrent/locally progressive pancreatic cancer. This study, however, was limited by its retrospective nature. This lead to prior radiation treatments not being standardized. Additionally, our series includes patients with locally PD who were treated with SBRT soon after EBRT as well as recurrent patients treated many months to years later. This adds heterogeneity to our population and may have an undetected effect on toxicity. Finally, our toxicity may be underrepresented due to possible uncaptured events associated with retrospective reviews.

## Conclusion

Locally progressive and recurrent pancreatic cancer is a therapeutic challenge, especially relative to local control. SBRT reirradiation is a feasible and tolerable treatment strategy for these patients. Due to the high precision SBRT affords, dose to critical structures, such as the small bowel, can be limited leading to reasonable levels of grade 3+ toxicity in patients with prior radiation. Future prospective studies should further define the role of SBRT in these patients.

## Ethics Statement

This project was approved by the University of Pittsburgh Institutional Review Board.

## Author Contributions

PS: data collection, data analysis, wrote and prepared manuscript. MB and DH: development of project, review and editing of final manuscript. Provided insight into how results relate to radiation oncology. HW: data analysis, ran all statistical tests, review and editing of final manuscript. SB: review and editing of final manuscript. Provided insight into how results relate to radiation oncology. NB: review and editing of final manuscript. Provided insight into how results relate to medical oncology. HZ: review and editing of final manuscript. Provided insight into how results relate to surgical oncology.

## Conflict of Interest Statement

The authors declare that the research was conducted in the absence of any commercial or financial relationships that could be construed as a potential conflict of interest.
