# Supplementary material for: Stereotactic Body Radiation Therapy for Locally Progressive and Recurrent Pancreatic Cancer after Prior Radiation
Source: Front Oncol. 2018 Mar 7;8:52. doi: 10.3389/fonc.2018.00052 (PMC5845878; doi:10.3389/fonc.2018.00052)
Supplement: Supplementary file 1 [file data_sheet_1.docx]

**Table S1: Univariate and Multivariate analysis of regional control.**

| **Factor** | **Hazard ration (95% confidence interval)** | **p-value** |
| --- | --- | --- |
| **Univariate Analysis** |  |  |
| age | 0.96 (0.86, 1.06) | 0.4266 |
| CA19-9 at diagnosis | 1.0003 (0.9994, 1.0012) | 0.4683 |
| Pre-SBRT CA19-9 | 1.0022 (0.9993, 1.0050) | 0.1440 |
| ­­­­­­­­­Post-SBRT CA19-9 | 1.0007 (0.9994, 1.0019) | 0.2935 |
| Change in CA19-9 | 1.003 (0.999, 1.008) | 0.1440 |
| SMAD4 mutated vs not | 1.17 (0.22, 6.20) | 0.8575 |
| Location: Heal vs Body | 27115874.22 (0.00, infinity) | 0.9983 |
| Location: Tail vs Body | 0.99 (0.00, infinity) | 1.0000 |
| Location: Uncinate vs Body | 0.99 (0.00, infinity) | 1.0000 |
| Location: Neck vs body | 67876652.97 (0.00, infinity) | 0.9982 |
| Location: multiple vs body | 0.99 (0.00, infinity) | 1.0000 |
| Location: Genu vs body | No data | - |
| Prior EBRT dose | 1.00 (0.90, 1.10) | 0.9696 |
| Modality: CyberKnife vs Triology | 2.82 (0.29, 27.15) | 0.3693 |
| Modality: Truebeam vs Triology | 0.00 (0.00, infinity)* | 0.9968 |
| Recurrent lesion being treated | 4.68 (0.49, 45.06) | 0.1817 |
| GTV volume | 1.01 (0.94, 1.09) | 0.7218 |
| PTV volume | 462.61 (0.00, infinity) | 0.9999 |
| Multiple fractions | 0.38 (0.04, 3.73) | 0.4096 |
| Chemo: Gemcitibine+Capcitabine vs Gemcitibine | 1.72 (0.16, 18.96) | 0.6594 |
| Chemo: FU based vs Gemcitibine | 3.11 (0.27, 35.23) | 0.3599 |
| gesurgery | 0.36 (0.05, 2.84) | 0.3324 |
| Dose | 0.95 (0.77, 1.18) | 0.6682 |

*The hazard ratio is 0 because the group defined by value 1 of the variable did not have a local progression event.

** The hazard ratio is very large because patients with low SB mean dose had censored data.

*** No multivariable model was found because all factors were non-significant in univariable models.

**Table S2: Univariate and Multivariate analysis of distant metastasis**.

| **Factor** | **Hazard ration (95% confidence interval)** | **p-value** |
| --- | --- | --- |
| **Univariate Analysis** |  |  |
| age | 0.97 (0.92, 1.01) | 0.1414 |
| CA19-9 at diagnosis | 0.9997 (0.9991, 1.0004) | 0.3920 |
| Pre-SBRT CA19-9 | 1.0013 (1.0002, 1.0024) | 0.0219 |
| ­­­­­­­­­Post-SBRT CA19-9 | 1.0001 (0.9993, 1.0009) | 0.8044 |
| Change in CA19-9 | 1.001 (0.998, 1.004) | 0.4595 |
| SMAD4 mutated vs not | 2.53 (0.51, 12.68) | 0.2582 |
| Location: Heal vs Body | 1.37 (0.17, 11.33) | 0.7686 |
| Location: Tail vs Body | 2.15 (0.13, 35.97) | 0.5952 |
| Location: Uncinate vs Body | 1.71 (0.17, 16.65) | 0.6463 |
| Location: Neck vs body | 0.00 (0.00, infinity)* | 0.9940 |
| Location: multiple vs body | 1.96 (0.17, 22.92) | 0.5934 |
| Location: Genu vs body | No data | - |
| Prior EBRT dose | 0.95 (0.90, 1.00) | 0.0314 |
| Modality: CyberKnife vs Triology | 0.73 (0.25, 2.12) | 0.5670 |
| Modality: Truebeam vs Triology | 0.34 (0.04, 2.77) | 0.3102 |
| Recurrent lesion being treated | 0.80 (0.26, 2.43) | 0.6899 |
| GTV volume | 1.042 (1.009, 1.077) | 0.0137 |
| PTV volume | 1.01 (0.98, 1.04) | 0.5511 |
| Multiple fractions | 0.86 (0.30, 2.45) | 0.7752 |
| Chemo: Gemcitibine+Capcitabine vs Gemcitibine | 1.18 (0.24, 5.76) | 0.8399 |
| Chemo: FU based vs Gemcitibine | 0.99 (0.12, 8.35) | 0.9916 |
| surgery | 0.74 (0.24, 2.28) | 0.6001 |
| Dose | 0.94 (0.84, 1.04) | 0.2486 |

*The hazard ratio is 0 because the group defined by value 1 of the variable did not have a local progression event.

** No multivariable model was found with the stepwise variable selection method.

**Table S3: Results of univariate logistic regression models for G3+ toxicity**.

| **Factor** | **Odds ratio (95% confidence interval)** | **p-value** |
| --- | --- | --- |
| GTV volume | 1.04 (0.98, 1.09) | 0.1872 |
| GTV max dose | 0.93 (0.75, 1.17) | 0.5492 |
| GTV min dose | 0.96 (0.79, 1.17) | 0.6861 |
| GTV mean dose | 0.92 (0.73, 1.17) | 0.5175 |
| PTV volume | 1.01 (0.96, 1.07) | 0.6856 |
| PTV max | 0.90 (0.70, 1.15) | 0.3955 |
| PTV min dose | 0.88 (0.68, 1.13) | 0.3173 |
| PTV mean dose | 0.83 (0.61, 1.12) | 0.2221 |
| Multiple fractions | 0.33 (0.03, 3.53) | 0.3618 |
| Differences in Dose/Fx schemes | 0.87 (0.70, 1.08) | 0.2112 |
| SB max dose | 0.85 (0.60, 1.20) | 0.3415 |
| SB mean dose | 25.91 (0.51, 1314.93) | 0.1043 |
